# Ultrasonographic diagnosis, classification, and treatment of cervical lymphatic malformation in paediatric patients: a retrospective study

**DOI:** 10.1186/s12887-020-02337-w

**Published:** 2020-09-19

**Authors:** Jiaoling Li, Wei Zhong, Xiuping Geng, Xiaofang Liu, Xiangxiang Zhang, Yurun Wang, Haibo Li

**Affiliations:** 1 Department of Ultrasound, Guangzhou Women and Children’s Medical Centre, Guangzhou Medical University, Jinsui Road 9, Guangzhou, 510623 China; 2 Neonatal Intensive Care Unit, Guangzhou Women and Children’s Medical Centre, Guangzhou Medical University, Jinsui Road 9, Guangzhou, 510623 China; 3 Department of Invasive Technology, Guangzhou Women and Children’s Medical Centre, Guangzhou Medical University, Jinsui Road 9, Guangzhou, 510623 China

**Keywords:** Cervical lymphatic malformation, Classification, Diagnosis, Magnetic resonance imaging, Treatment, Ultrasound

## Abstract

**Background:**

To explore the imaging features, key diagnostic points, classification, treatment, and prognosis of cervical lymphatic malformation.

**Methods:**

Overall, 320 patients diagnosed with cervical lymphatic malformation were retrospectively analysed in our hospital between 1 January 2014 and 31 December 2017. Imaging modalities included colour Doppler ultrasound, magnetic resonance imaging, and contrast-enhanced computed tomography. Cervical lymphatic malformations were classified by cyst diameter. Treatments included interventional therapy, surgery, and expectant treatment.

**Results:**

Cervical lymphatic malformation was identified in 320 of 1192 patients with lymphatic malformation. Four were excluded due to misdiagnosis by ultrasonography. Cervical lymphatic malformation was classified as mixed, macrocystic, and microcystic in 184 (57.5%), 117 (36.56%), and 19 (5.94%) patients, respectively. Sixty-four (20%), ten (3.12%), seven (2.19%), and three (0.94%) patients experienced intracystic haemorrhage, infection, concurrent intracystic haemorrhage and infection, and calcification, respectively. Among 260 (81.25%) patients who underwent interventional sclerotherapy, 163 (50.94%) received it once and 96 (30%) received it two or more times. Twenty-eight (8.75%), five (1.56%), and 27 (8.44%) patients underwent surgical resection, interventional sclerotherapy plus surgery, and expectant management, respectively.

**Conclusions:**

Ultrasonography is useful for diagnosing definite cervical lymphatic malformation. Interventional therapy is the first choice for children with confirmed cervical lymphatic malformation.

## Background

Cervical lymphatic malformation is the most common lymphatic malformation and cervical vascular malformation in children [1–3], with an incidence of 1.2–2.8 per 1000 individuals. It develops prenatally and can be diagnosed by prenatal ultrasonography in 50–65% of patients but by postnatal ultrasonography in 90% of patients. It is not typically diagnosed in adults. Surgical and other trauma can destroy the lymphatic drainage system, leading to acquired lymphatic malformation; other causes are also possible [4, 5]. Its clinical manifestations depend on the infiltration location and growth rate, and approximately 15–70% of patients have mild symptoms [6–8]. Ultrasonographic diagnosis and differential diagnosis are based on intracystic fluid echoic, the thickness of cyst wall, and the cyst location; additionally, medical history and clinical manifestations are helpful for diagnosis.

Cervical lymphatic malformation can present as unilocular or multilocular cysts, and the cyst diameter varies from few millimetres to several centimetres [9]. Therefore, it can be classified as macrocystic, microcystic, or a mixed type according to the cyst diameter. Although cervical lymphatic malformations are benign, they can invade adjacent critical structures, which increases the difficulty of surgical resection and the risk of postoperative recurrence. The malformations may also threaten the function of adjacent or affected tissues, organs, and as a result, appropriate treatment may be avoided [10, 11]. Cervical lymphatic malformations can predispose an individual to complications such as haemorrhage or infection, which further affect respiratory function and endanger life [12–14].

Multiple imaging methods should be used to classify and make differential diagnoses of cervical lymphatic malformations, monitor lesion progression, and assess the relationship of the lesion with adjacent structures to guide the optimal treatment method selection [15, 16], reduce complications and recurrence, and avoid injury.

The aim of this retrospective single-centre study was to explore the ultrasonographic characteristics of patients with cervical lymphatic malformations in southern China and summarise key points of their diagnosis, differential diagnosis, classification, and treatment selection as well as analyse the causes of misdiagnoses and missed diagnoses.

## Methods

This study was approved by the Ethics Review Board of the GZ Women and Children’s Medical Centre (4AE4237E-7889-4275-95E5-8F469A5C9188). Parents of the children provided informed consent for ultrasonography and treatment options. Written consent was obtained from parents of participants in this study.

### Patient selection

Records in the large data centre at our hospital were searched and data on patients diagnosed with lymphatic malformation or cervical lymphatic malformation between 1 January 2014 and 31 December 2017 were collected. We used “lymphatic malformation” and “cervical lymphatic malformation” as keywords in the search. Three hundred and twenty-two patients were initially identified; four were excluded due to misdiagnosis by ultrasonography, and two previously excluded patients were included: one was due to a missed diagnosis and the other developed cervical lymphatic malformation after pyriform cyst surgery. Therefore, 320 patients with confirmed cervical lymphatic malformation were included in the analysis. The mean age of the patients was 2 years and 29 days (range, 1 day to 14 years). Thirty-five patients were prenatally identified as having cervical lymphatic malformation. Twenty-seven patients underwent a follow-up ultrasonographic assessment once every 2 months at > 6 months after diagnosis; 260 patients underwent intraoperative puncture result assessment; 5 patients, underwent intraoperative puncture and postoperative pathological confirmation; and 28 patients, postoperative pathological confirmation of the diagnosis (regarded as the gold standard to determine the sensitivity of ultrasonographic diagnosis).

### Imaging, diagnosis, and classification

A high-resolution ultrasound system, equipped with a 4–15 MHz transducer (Acuson S2000; Siemens, Hamberg, Germany), was used to diagnose cervical lymphatic malformation. Patients in whom the relationship between the lesion and adjacent structures was not clear by ultrasonography because of individual cysts < 1 mm in diameter underwent further evaluation by magnetic resonance imaging (MRI) (Signa; GE Medical Systems, Milwaukee, WI, USA) or contrast-enhanced computed tomography (CT). All patients underwent colour Doppler and power Doppler ultrasonography. Ultrasonographic diagnosis and differential diagnosis were based on the intracystic fluid echo, thickness of cyst wall, cyst location, and colour flow signal distribution. The macrocystic (cysts > 1 cm in diameter), microcystic (individual cysts < 1 cm in diameter), and mixed types were classified according to the cyst diameter. Cervical lymphatic malformation was diagnosed with haemorrhage, infection, and/or calcification according to increased cyst diameter (compared with prior size) and intracyst fine spot echo, local pain, increased temperature and abnormal blood test results, and calcified plaque in cyst before treatment, respectively.

### Treatments

Patients were treated with interventional sclerotherapy, surgery, or surgery combined with interventional therapy.

The principles and methods of interventional sclerotherapy [17] (using bleomycin at the recommended dose of 0.5 mg/kg in an aqueous solution of 1.5 mg/mL) were as follows: (1) to prevent the side effects of sclerosing agents in infants, patients were > 6 months old unless symptoms of oppression occurred; (2) individual cysts had to be > 1 cm in diameter; (3) imaging findings should suggest infiltration of the lesion into adjacent critical structures; (4) Patients with cervical lymphatic malformation and patients complicated by infection first had to undergo anti-inflammatory treatment for 3–5 days before a sclerosing agent will be injected into the lesion; (5) For abscess formation complicating cervical lymphatic malformation, ultrasound-guided puncture and drainage was administered to patients followed by a simultaneous injection of a sclerosing agent; (6) cervical lymphatic malformation patients with haemorrhage first had to undergo haemostasis, after which a sclerosing agent was injected into the lesion; and (7) for patients without complications, a sclerosing agent had to be injected into the lesion or they had to undergo follow-up ultrasonography.

If the lesion did not progress, expectant treatment (namely routine observation) continued. For patients in whom the lesion increased or hardened (compared with prior size or texture), interventional sclerotherapy or surgery was performed, with routine blood tests, screening of the coagulation status, and liver function tests. For all patients who underwent interventional sclerotherapy, the puncture liquid was smeared for definite diagnosis, and a contrast medium was injected to determine the size of the cyst. If the cysts were interconnected, we chose the dose of the contrast medium that excluded lymphovascular malformations.

Patients with individual cysts < 1 cm in diameter, or patients with macrocystic or mixed types but with lesion size more than 4 cm, underwent surgical treatment, and the determinant for surgery combined with interventional therapy was if lesion size was more than 4 cm in diameter. The interventional therapy targets residual lesions to prevent toxic side effects caused by excessive use of sclerosing agents.

## Results

Cervical lymphatic malformation was identified in 320 of the 1192 patients with lymphatic malformation examined during our observation period with 189 males and 131 females (1.4:1 male-to-female ratio). All 320 patients had undergone further evaluation by colour Doppler ultrasonography and power Doppler ultrasonography, with 43 patients further assessed by MRI and 55 by contrast-enhanced CT. Details regarding the types of complications and medical management are summarised in Tables 1 and 2. Complications occurred in 84 patients (26.25%); of them, 64 (20%) experienced haemorrhage, 10 (3.12%) developed infections, 7 (2.19%) had concurrent haemorrhage and infection, and 3 (0.94%) exhibited calcification. Of the 260 patients (81.25%) who underwent sclerotherapy, 163 (50.94%) underwent bleomycin sclerotherapy once and 96 (30%) underwent bleomycin sclerotherapy two or more times, with one patient (0.31%) receiving seven rounds. Twenty-eight patients (8.75%) received surgical treatment and five (1.56%) received concurrent surgical treatment and bleomycin sclerotherapy. Expectant management was provided to the other 27 patients (8.44%).

**Table 1:** Summary of ultrasonographic typing and complications of 320 patients of cervical lymphatic malformation (case (%))

Typing	Patient number	Simple cervical lymphatic malformation	Haemorrhage	Infection	Haemorrhage and infection	Calcification
Mixed type	184	143	32	3	3	3
Macrocystic type	117	78	31	5	3	0
Microcystic type	19	15	1	2	1	0
Total	320	236 (73.75)	64 (20)	10 (3.12)	7 (2.19)	3 (0.94)

**Table 2:** Summary of ultrasonographic typing and treatments of 320 patients of cervical lymphatic malformation (case (%))

Typing	Patient number	Interventional therapy (once)	Interventional therapy (twice or more)	Operation	Operation combined with interventional therapy	Expectant treatment
Mixed type	184	85	62	16	4	17
Macrocystic type	117	69	32	11	0	5
Microcystic type	19	9	3	1	1	5
Total	320	163 (50.94)	97 (30.31)	28 (8.75)	5 (1.56)	27 (8.44)

**Fig. 1:**
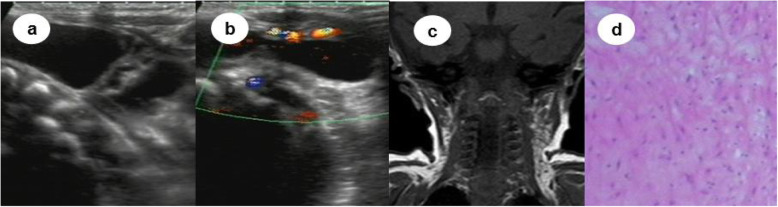
Patient with a mixed cervical lymphatic malformation who underwent operation combined with sclerotherapy

**Fig. 2:**
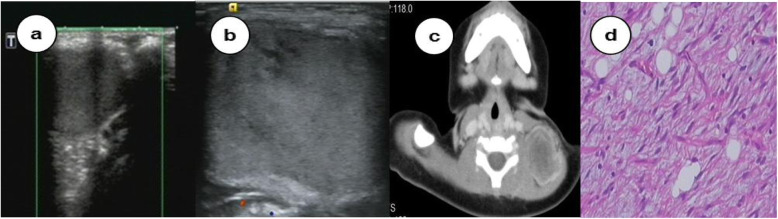
A patient with a lipoblastoma misdiagnosed as a cervical lymphatic malformation complicated with haemorrhage

The ultrasonographic features and treatments of one representative patient with cervical lymphatic malformation and those of the patient misdiagnosed with lipoblastoma are shown in Figs. 1 and 2. Of the 320 patients, only 19 (5.94%) showed sparse flow signals on the cyst wall and septations.

Among the 28 patients who underwent surgical treatment, only one experienced chylous fistula. Of the 260 patients who underwent bleomycin sclerotherapy, only one developed an infection. All lesions gradually resolved, with the longest taking 2 years. Two patients with pyriform cysts, one patient with a dermoid cyst, and one patient with lipoblastoma were misdiagnosed with cervical lymphatic malformation, whereas one patient with cervical lymphatic malformation was misdiagnosed as having a pyriform cyst.

## Discussion

### Main findings

In this retrospective study of 320 patients, including one patient who developed cervical lymphatic malformation after pyriform cyst surgery and one patient who was misdiagnosed by ultrasonography, the incidence of cervical lymphatic malformation complications was 26.25% before treatment. One patient developed a chylous fistula and another developed an infection; the total success rate of treatment was 99.38%.

### Interpretation

Cervical lymphatic malformation, a benign congenital disease, is caused by non-transportation of the lymphatic and venous systems, abnormal hyperplasia of the lymphatic epithelium, or lymphatic obstruction. Additionally, surgical trauma to the neck can destroy the lymphatic drainage system, resulting in acquired cervical lymphatic malformation [18]. In this study, it was found that cervical lymphatic malformation also developed as a complication of pyriform cyst surgery.

Lymphatic malformations can occur in all areas of the body, but 75% occur in the neck [19, 20]. In this study, cervical lymphatic malformation was found to constitute 26.85% of all lymphatic malformation cases (320/1192). This rate is lower than that observed previously, where foetal cervical lymphatic malformation comprised 63.29% of all lymphatic malformation cases [2, 19]. This may be because more pregnancies were terminated due to cervical lymphatic malformations accompanied by severe structural or chromosomal abnormalities in the foetus, or because some lesions subsided naturally [21]. Two-dimensional ultrasonography showed that most cervical lymphatic malformations were multilocular cysts; large cysts had a fine separation, and small cysts had a coarse separation. Few lymphatic malformations were unilocular cysts with thin walls and good sound transmission. The boundaries of the lesions were unclear in the microcystic and mixed types but clear in the macrocystic type. Therefore, classification may help in treatment selection.

The diagnosis and classification of cervical lymphatic malformations are established through clinical manifestations and imaging data. However, cervical lymphatic malformations can invade adjacent structures, and ultrasonography is limited in resolving the boundaries of cysts; MRI and CT scan have better tissue resolution. In our study, 43 and 55 patients underwent MRI and CT scan, respectively. When a cervical lymphatic malformation is complicated by intracystic haemorrhage or infection, fine spot echoes can be observed in the cyst [22]. With complications of cystic wall fibrosis or hardening, stripe hyperechogenicity can be observed in the cyst wall [23]. In this study, 2.19% patients developed concurrent intracystic haemorrhage and infection, 20% developed intracystic haemorrhage, 3.12% developed an infection, and 0.94% developed calcification. No patients in our previous analysis of foetal lymphatic malformation experienced these complications. These complications may be related to friction and trauma due to discomfort caused by the neck mass in children.

Differentiating cervical lymphatic malformations from other conditions is essential for proper treatment. Colour and power Doppler ultrasonography do not show flow signals in the wall and septations of most cervical lymphatic malformations. Of the 320 patients with cervical lymphatic malformations in our study, 19 showed sparse flow signals in the wall and septations of the masses during colour and Doppler ultrasonography. Therefore, this feature can be used to differentiate cervical lymphatic malformations from cervical haemangiomas, which shows rich flow signals. Unilocular cervical lymphatic malformations should be differentiated from pyriform cysts, which are generally located anterolateral to the trachea and have thicker walls [2, 3]. In this study, two patients with pyriform cyst were misdiagnosed with cervical lymphatic malformations, and one patient with a cervical lymphatic malformation was misdiagnosed with a pyriform cyst. When a cervical lymphatic malformation is complicated by haemorrhage, infection, or calcification, it should be differentiated from a dermoid cyst. Dermoid cysts are most commonly located in the submental part of the midline of the neck, above the hyoid bone, and have a thicker wall than cervical lymphatic malformation [24]. On imaging, internal echoic patterns of dermoid cysts are generally low and turbid, the pattern of distribution is scattered, and real-time ultrasonography displays fine spot rolling and reflection stripes in the envelope [25]. In this study, one patient with dermoid cyst was misdiagnosed with a cervical lymphatic malformation. Moreover, cervical lymphatic malformations should be differentiated from cervical lipoblastoma, which is common in infants < 3 years old. These masses occur separately and have a medium-to-soft texture, clear boundaries, and good mobility. Most lesions are located under the skin, presenting as slightly hyperechoic masses with hyperechoic septations inside. Colour Doppler ultrasonography may reveal low-speed strip colour Doppler signals in and around the tumour [26]. Therefore, according to its clinical features and the echoic pattern, location, and distribution of blood flow, differentiating cervical lymphatic malformations from other conditions can be easy.

Although 1.6–16% of lymphatic malformations subside naturally and 15–70% have mild symptoms and only need outpatient follow-up [7], approximately 50% of cervical lymphatic malformations, especially those complicated by haemorrhage and infection, cause compression and distortion of important adjacent organs, affecting breathing, swallowing, and making sounds, and can be life-threatening. Therefore, surgery, radiotherapy, or interventional therapy must be carried out. However, surgery may damage adjacent blood vessels and nerves, cause hematoma, and affect appearance. Moreover, the incidence of complications from surgery is 19–33%, the postoperative recurrence rate is 53%, and the mortality rate is 6% [7, 27]. Therefore, interventional sclerotherapy is a better choice for recurrent and surgically unresectable lesions, reducing tumour volume before surgery, reducing injury, and improving aesthetic appearance [7, 28]. Sclerotherapy has thus become the primary treatment for cervical lymphatic malformations; however, it has side effects such as metabolic acidosis, hyperhaemoglobinaemia, and cellulitis [29]. Therefore, patients with large-volume or multilocular cysts should undergo sclerotherapy in stages or for residual lesions after surgical resection to avoid toxic side effects. In this study, patients received treatment according to their condition; most patients underwent sclerotherapy once, and few patients underwent sclerotherapy two or more times. Several patients underwent surgical resection or concurrent surgical resection and sclerotherapy. Expectant management was provided for patients with progressive reduction of lesions. The total success rate of treatment was 99.38%. All of the lesions eventually disappeared, with the longest regression time being 2 years.

### Strengths and limitations

The strength of this study is its sample size; to the best of our knowledge, this is the largest study to date on cervical lymphatic malformations in southern China.

Our study has some limitations. First, whether the occurrence of an isolated cervical lymphatic malformation is related to gender is unclear. Among 320 patients with cervical lymphatic malformations, the male-to-female ratio was 1.4:1. A multicentre study with a large sample size would clarify this association. Second, this study did not stage cervical lymphatic malformations, and treatment methods used for the three cervical lymphatic malformation types were not compared. These aspects should be explored in a subsequent study.

## Conclusion

In conclusion, our results demonstrated that accurate ultrasonographic diagnosis and classification of cervical lymphatic malformations may provide a basis for selecting its most appropriate treatment method. For foetuses with cervical lymphatic malformations, strengthening prenatal ultrasonographic monitoring and understanding the extent of lesion progression, tracheal compression, and distortion are vital. Multidisciplinary teams should thus be involved in comprehensively selecting delivery modes and performing timely delivery. During the neonatal period or childhood, multiple imaging methods, including ultrasonography, MRI, and CT, should be used to monitor the relationship of the lesion with the adjacent structures, thus allowing surgeons to select the most appropriate approach.

## Supplementary information

**Additional file 1:.**

## Data Availability

All data generated or analyzed during this study are included in this article and could be found in Additional file 1. (Additional file 1: Description of the data and material of 320 cases with cervical lymphatic malformation).

## Abbreviations

MRI: Magnetic resonance imaging
CT: Computed tomography
